# Environmental toxin acrolein alters levels of endogenous lipids, including TRP agonists: A potential mechanism for headache driven by TRPA1 activation

**DOI:** 10.1016/j.ynpai.2017.03.001

**Published:** 2017-04-01

**Authors:** Emma Leishman, Phillip E. Kunkler, Meera Manchanda, Kishan Sangani, Jordyn M. Stuart, Gerry S. Oxford, Joyce H. Hurley, Heather B. Bradshaw

**Affiliations:** a Department of Psychological and Brain Sciences, Indiana University, 1101 East 10^th^ Street, Bloomington, IN 47405, USA; b Stark Neurosciences Institute, Indiana University School of Medicine, 320 West 15^th^ Street, Indianapolis, IN 46202, USA

**Keywords:** 2-AG, 2-arachidonyl glycerol, AA, arachidonic acid, AEA, *N*-arachidonoyl ethanolamine, A-GABA, *N*-arachidonoyl GABA, A-Phe, *N*-arachidonoyl phenylalanine, A-Ser, *N*-arachidonoyl serine, A-Taur, *N*-arachidonoyl taurine, A-Tyr, *N*-arachidonoyl tyrosine, CER, cerebellum, CNS, central nervous system, d_8_NAGly, deuterium labeled *N*-arachidonoyl glycine, DAG, diacylglycerol, DEA, *N*-docosahexaenoyl ethanolamine, D-Gly, *N*-docosahexaenoyl glycine, DMEM, Dulbecco’s Modified Eagle’s Medium, eCB, endogenous cannabinoid, FAAH, fatty acid amide hydrolase, FBS, fetal bovine serum, HPLC/MS/MS, high pressure liquid chromatography coupled to tandem mass spectrometry, KO, knockout, LEA, *N*-linoleoyl ethanolamine, L-Gly, *N*-linoleoyl glycine, MAGL, monoacylglycerol lipase, NAE, *N*-acyl ethanolamine, NAGly, *N*-arachidonoyl glycine, OEA, *N*-oleoyl ethanolamine, O-Gly, *N*-oleoyl glycine, O-Phe, *N*-oleoyl phenylalanine, O-Val, *N*-oleoyl valine, PEA, *N*-palmitoyl ethanolamine, PG, prostaglandin, PLC, phospholipase C, P-Phe, *N*-palmitoyl phenylalanine, S-Gly, *N*-stearoyl glycine, S-Phe, *N*-stearoyl phenylalanine, TG, trigeminal ganglia, TNC, trigeminal nucleus caudalis, TRP, transient receptor potential, TRPA1, TRP ankyrin 1, TRPV1, TRP vanilloid 1, TVS, trigeminovascular system, Lipidomics, Endogenous cannabinoid, TRPA1, TRPV1, Lipoamine, Acrolein, Migraine

## Abstract

Exposure to airborne toxins can trigger headaches, but the mechanisms are not well understood. Some environmental toxins, such as acrolein, activate transient receptor potential ankyrin 1 (TRPA1), a receptor involved in pain sensation that is highly expressed in the trigeminovascular system. It has been shown in rat models that repeated exposure to acrolein induces trigeminovascular sensitization to both TRPA1 and TRP vanilloid 1 (TRPV1) agonists, a phenomenon linked to headache. In this study, we test the hypothesis that the sensitization of trigeminovascular responses in rats after acrolein exposure via inhalation is associated with changes in levels of endogenous lipids, including TRPV1 agonists, in the trigeminal ganglia, trigeminal nucleus, and cerebellum. Lipidomics analysis of 80 lipids was performed on each tissue after acute acrolein, chronic acrolein, or room air control. Both acute and chronic acrolein exposure drove widespread alterations in lipid levels. After chronic acrolein exposure, levels of all 6 N-acyl ethanolamines in the screening library, including the endogenous cannabinoid and TRPV1 agonist, *N*-arachidonoyl ethanolamine, were elevated in trigeminal tissue and in the cerebellum. This increase in TRPV1 ligands by acrolein exposure may indicate further downstream signaling, in that we also show here that a combination of these TRPV1 endogenous agonists increases the potency of the individual ligands in TRPV1-HEK cells. In addition to these TRPV1 agonists, 3 TRPV3 antagonists, 4 TRPV4 agonists, and 25 orphan lipids were up and down regulated after acrolein exposure. These data support the hypothesis that lipid signaling may represent a mechanism by which repeated exposure to the TRPA1 agonist and environmental toxin, acrolein, drives trigeminovascular sensitization.

## 1 Introduction

### 1.1 TRPA1 agonists activate the trigeminovascular system

Headache is an almost universal experience. For people with migraines, these headaches become chronic and disabling (Benemei et al., 2014, Oxford and Hurley, 2013). Migraine is an episodic disorder, meaning that symptoms are not always present (Benemei et al., 2014). One of the most common triggers for migraine episodes is exposure to airborne toxins in air pollution (Friedman et al., 2009); however, the mechanism is largely unknown (Kunkler et al., 2015). Activation of the trigeminovascular system (TVS), the trigeminal innervation of cranial vasculature (Goadsby and Edvinsson, 1993), is hypothesized to underlie headache pain (Kunkler et al., 2015). Cell bodies of trigeminal nerve fibers are located in the trigeminal ganglia (TG) Goadsby and Edvinsson, 1993, which projects centrally to the trigeminal nucleus caudalis (TNC) in the brainstem, where sensory signals are projected onto thalamo-cortical pathways (Eftekhari and Edvinsson, 2011). With repeated TVS activations, sensitization can occur, which may underlie the phenomenon of heightened headache pain after repeated chemical exposure (Kunkler et al., 2015). Some clues as to how inhaled irritants sensitize the TVS may derive from their receptor target: transient receptor potential ankyrin 1 (TRPA1). TRPA1 is expressed in sensory neurons, and topical application of TRPA1 agonists produces acute pain (Jordt et al., 2004). The TRPA1 agonist acrolein (Supplementary Fig. 1) is an irritant and a major component of air pollution (Feng et al., 2006, Brone et al., 2008). Molecular mechanisms of inflammatory pain and hypersensitization of the TVS caused by irritants like acrolein depend, in part, on TRPA1 activity (Kunkler et al., 2011, Bautista et al., 2006).

### 1.2 Lipoamines as endogenous activators of TRP channels

Previous studies suggest co-localization and significant cross-talk between TRPA1 and TRP vanilloid 1 (TRPV1) signaling Salas et al., 2009. Ablation of TRPV1 in the TG inhibits TVS activation driven by TRPA1 agonists (Kunkler et al., 2014). Many endogenous TRPV ligands are lipoamines, which are endogenous lipids that are conjugations of fatty acids and amines (Supplemental Fig. 2) (Bradshaw et al., 2012, Milman et al., 2006, Smoum et al., 2010, Tortoriello et al., 2013). As one of the most studied lipoamines, the endogenous cannabinoid (eCB) *N*-arachidonoyl ethanolamine (AEA) activates TRPV1 as well as cannabinoid receptors (Zygmunt et al., 1999). Additional lipoamines also act as ligands at TRPV1: *N*-oleoyl ethanolamine (OEA) Ahern, 2003 Movahed et al., 2005, *N*-linoleoyl ethanolamine (LEA), and *N*-docosahexaenoyl ethanolamine (DEA) Raboune et al., 2014, and certain lipoamines derived from arachidonic acid (AA) such as *N*-arachidonoyl GABA (A-GABA) Raboune et al., 2014 and *N*-arachidonoyl taurine (A-Taur) Saghatelian et al., 2006. Recently, we showed that many of these endogenous TRPV1 agonists were upregulated in the CNS in a model of inflammatory pain (Raboune et al., 2014), including in brain regions distant from the site of injury. To date, there are no data on how lipoamines and other small molecule lipids may also play a role in headache pain.

To test the hypothesis that acute and chronic exposure of the toxin acrolein alters the central nervous system (CNS) lipidome, we performed targeted lipidomics screens of the TG, TNC and a control tissue, the cerebellum (CER), from rats exposed to either acrolein or room air. Analyzing AEA, 73 of its lipoamine structural analogs, 2-arachidonoyl glycerol (2-AG), its 2-acyl glycerol structural analogs, 3 prostaglandins (PGs), and free fatty acids through targeted HPLC/MS/MS we were able to determine how this lipidome is modified. These data illustrated at least 2 patterns of increases in TRPV1 lipoamine agonists as well as 2-acyl glycerols. In that these TRPV1 agonists increased together in these tissues, we tested the hypothesis that their activity at TRPV1 would be more potent as a group than their activity alone using a TRPV1-HEK expression system and calcium imaging assays. We next tested the hypothesis that TRPV1 activity would drive subsequent lipid production by performing lipidomics screens on these TRPV1-HEK cells after activation by capsaicin showing that TRPV1 activity promotes an increase in 2-acyl glycerols but not lipoamines. Together, these experiments provide evidence that acrolein exposure drives changes in CNS lipids that support a working hypothesis that both TRPA1 and TRPV1 activity play a role in the headache induced by acrolein exposure.

## 2 Methods

### 2.1 Rats, acrolein exposure and tissue collection

All animal procedures were approved by the Institutional Animal Care and Use Committee of Indiana University School of Medicine and were in accordance with the ethics guidelines set by the International Association for the Study of Pain (Zimmermann, 1983) and with ARRIVE guidelines. All efforts were made to minimize animal suffering and to reduce the number of animals used. For these experiments, 24 adult male (170–250 g) Sprague-Dawley rats (Harlan, Indianapolis IN) were used: 12 were assigned to the acute paradigm and 12 were assigned to the chronic paradigm. Rats were housed in pairs with food and water available ad libitum, and were kept on a standard light/dark cycle.

Acrolein exposure took place as described in Kunkler and colleagues (Kunkler et al., 2015). Rats were placed in 5.5 L temperature and humidity controlled inhalation chambers (Braintree Scientific, Braintree, MA). In the acute exposure paradigm, 6 rats were individually placed in the chamber for 4 h while acrolein gas (Air Liquide, Plumsteadville, PA) was mixed with room air at a concentration of 0.3 ppm and flow rate of 1.5 L/min. This dose was chosen because it is the upper limit for short-term exposure recommended by OHSA and does not have detectable harmful effects. A control group of 6 animals was exposed individually over an identical period to normal room air only, at a flow rate of 1.5 L/min. To minimize cross-contamination, separate chambers and tubing were used for the 2 groups. To study the effects of chronic exposure, 6 additional rats were exposed to acrolein (0.3 ppm) for 4 h daily for 4 consecutive days and 6 rats were individually placed in the room air chamber for 4 h a day for 4 consecutive days. Acrolein exposure for each animal was monitored using badges placed in the chamber (Advanced Chemical Sensors, Boca Raton, FL).

After each type of exposure paradigm, rats were sacrificed via decapitation, either immediately after the acute 4 h exposure or 24 h after the last chronic exposure session. Following removal of the brain, the left TG was removed from the skull. The TNC was rapidly dissected from tissue corresponding to the lower brainstem through C2 cervical segments of the dorsal spinal cord and tissue from the right cerebellar hemisphere was dissected for CER analysis. Tissues were frozen on dry ice and stored at −80 °C before being processed for lipid extraction.

### 2.2 Lipid extraction and high pressure liquid chromatography-coupled tandem mass spectrometry (HPLC/MS/MS)

#### 2.2.1 Trigeminal ganglia, trigeminal nucleus, and cerebellum lipid partial purification

Tissue extracts were performed as previously described (Tortoriello et al., 2013, Leishman et al., 2016a, Leishman et al., 2016b, Tan et al., 2006, Stuart et al., 2013). In brief, frozen tissue were weighed, placed in 50 times their weights of methanol, and spiked with 500 picomols deuterium-labeled *N*-arachidonoyl glycine (d_8_NAGly; Cayman Chemical, Ann Arbor, MI). Samples were placed on ice in darkness for 2 h then individually homogenized and centrifuged at 19,000*g* for 20 min at 20 °C. Supernatants were decanted and diluted with HPLC water (purified in house) to make a 75% water, 25% supernatant solution. Partial purification was achieved using C-18 solid phase extraction columns (Agilent, Palo Alto, CA). A series of 4 elutions with 1.5 mL of 60%, 75%, 85%, and 100% methanol were collected for analysis (Leishman et al., 2016a, Stuart et al., 2013). Vials of eluants were stored at −80 °C until they were ready for analysis.

#### 2.2.2 TRPV1-HEK cell lipid partial purification

hTRPV1-HEK, HEK293 cells stably transfected with human TRPV1, which were a kind gift from Merck (Whitehouse Station, NJ) were grown and maintained under standard cell culture conditions, as described in Section 2.4. The cells were split into 10 T-25 cm^2^ flasks (Sigma Aldrich, St. Louis, MO) and allowed to incubate for 24–48 h in 10% fetal bovine serum (FBS) Dulbecco’s Modified Eagle’s Medium (DMEM). Upon reaching ∼75% confluence, the flasks were divided into two treatment groups to be stimulated for one minute with either 10 µM capsaicin or 0.1% DMSO (serving as vehicle). Prior to stimulation, the cells were washed twice with HEPES-Tyrode buffer and were then incubated at 37 °C in 4 mL HEPES buffer for 45 min, allowing the cells to equilibrate. The buffer was then aspirated off and 4 mL buffer containing 10 µM of capsaicin or vehicle was added individually to each flask and the cells were incubated in this solution for 1 min. After the stimulation period, buffer was transferred from each flask into a corresponding 15 mL centrifuge tube, 2 mL of 100% HPLC-grade methanol added to each flask for 2 min and the cells harvested from the flasks using a scraper and added to the corresponding 15 mL centrifuge tube. The flask surfaces were finally rinsed with an additional 2 mL of methanol to collect any remaining cells. The cell solutions were spiked with 10 µM d_8_NAGly and centrifuged at 3000 rpm for 15 min at 24 °C. Partial purification of the supernatant was performed identically to the tissue samples described in Section 2.2.1.

#### 2.2.3 HPLC/MS/MS

Elutions were analyzed using an Applied Biosystems API 3000 triple quadrupole mass spectrometer with electrospray ionization (Foster City, CA) as previously described (Tortoriello et al., 2013, Leishman et al., 2016b). 20 µL from each elution were chromatographed using XDB-C18 reversed phase HPLC analytical column (Agilent) and optimized mobile phase gradients. Mobile phase A: 20% methanol, 80% water (v/v) and 1 mM ammonium acetate (Sigma Aldrich). Mobile phase B: 100% methanol, 1 mM ammonium acetate. Two Shimadzu 10ADvp pumps (Columbia, MD) provided the pressure for gradient elution. Every method run began with 0% mobile phase B, reached a state of 100% mobile phase B flowing at 0.2 mL per minute, and gradually returned to 0% mobile phase B.

### 2.3 Data analysis and statistical procedures for lipid analyses

Levels of each compound were determined by running each sample using a multiple reactions monitoring method tailored for each group of structurally similar compounds (Supplemental Fig. 3) as previously described (Leishman et al., 2016b). HPLC/MS/MS data was analyzed using Analyst software (Applied Biosystems, Framingham, MA) as previously described (Tortoriello et al., 2013, Leishman et al., 2016a, Leishman et al., 2016b, Tan et al., 2006, Stuart et al., 2013). Chromatograms were generated by determining the retention time of analytes with a [M−1] or [M+1] parent peak and a fragmentation peak corresponding to the programmed values. The retention time was then compared to the retention time of a standard for the suspected compound. If the retention times matched, then the concentration of the compound was determined by calculating the area under the curve for the unknown and comparing it to the calibration curve obtained from the standards. Therefore, unknown lipids are matched to known standards according to retention time from the analytical column and their mass fingerprint.

Extraction efficiency was calculated with the d_8_NAGly spiked recovery vial as a standard as done previously (Tortoriello et al., 2013, Leishman et al., 2016a, Leishman et al., 2016b, Raboune et al., 2014, Tan et al., 2006, Stuart et al., 2013). For each individual lipid in the TG, TNC and CER, concentrations in moles per gram adjusted for percent recovery from the acrolein treated animals were compared to control concentrations using a one-way ANOVA. Statistical tests were carried out using SPSS Statistics (IBM, Armonk, NY) and significance was defined as p < 0.05 and p < 0.10.

### 2.4 Calcium imaging reagents

hTRPV1-HEK cells were grown and maintained under standard cell culture conditions in high-glucose DMEM, supplemented with 10% FBS and 1% penicillin/streptomycin (Thermo Fisher Scientific, Waltham, MA). HEPES-Tyrode buffer solution was prepared with HEPES 0.025 mol, NaCl 0.140 mol, KCl 0.0027 mol, CaCl_2_ 0.0018 mol, MgCl_2_ 0.0005 mol, NaH_2_PO_4_ and D-glucose 0.05 mol (Sigma Aldrich). Fura-2AM (Invitrogen, Carlsbad, CA) was stored at -20 °C and protected from light sources. A 20% (w/v) Pluronic F-127 (Sigma Aldrich) solution was prepared in-house in >99% DMSO and stored at room temperature for up to 1 h before being transferred to cells at the time of assay. Stock solutions of the *N-*acyl ethanolamines (NAEs; Cayman Chemical) and capsaicin (Sigma Aldrich) were prepared in-house in 100% ethanol and stored at −80 °C. Solutions of appropriate molarities were made immediately before experimentation, in >99% DMSO.

### 2.5 Calcium imaging

All cellular preparation and assay parameters were identical to those previously described (Raboune et al., 2014). In brief, cells were incubated with Fura-2AM/Pluronic for 30 min then washed and equilibrated in HEPES buffer for 30 min prior to measurements using a FlexStation II (Molecular Devices, Sunnyvale, CA). hTRPV1-HEK cells were challenged with target compounds with the configuration illustrated in Supplemental Fig. 4.

### 2.6 Calcium imaging statistical analyses

Dose-response curves for each experimental combination were generated using either a sigmoidal four parameter logistic equation with GraphPad Prism 6.0 (GraphPad Software, La Jolla, CA). EC_50_ values were calculated using nonlinear regression analyses (GraphPad Prism). If an asymptote was not observed for a given dose-response curves, the EC_50_ was calculated based on trends seen between the six concentrations tested. This type of analysis was justified in that the intent of the comparisons is not to determine the absolute EC_50_, but the relative EC_50_ given the identical context.

## 3 Results

### 3.1 Signal detection of lipids in screening library

Of the 71 lipoamines in our screening library, over 50 were detected in the TG, TNC, and CER in both control and acrolein treated rats. Furthermore, the same subset of lipids was detected in both acute and chronic paradigms in each tissue. Similar numbers of lipids were detected in the TG (64 lipids – 80% of the lipids in the screening library), in the TNC (63 lipids – 79%), and in the CER (62 lipids – 78%). This level of analysis constitutes ∼6000 data points; therefore, the detailed list of levels and the statistical analyses of analytes detected in each of the 3 tissues assayed from acute acrolein rats, chronic acrolein rats, and the room air controls are available in the Supplemental Data section.

### 3.2 Overall effects of acrolein exposure on the lipidome

**Fig. 1:**
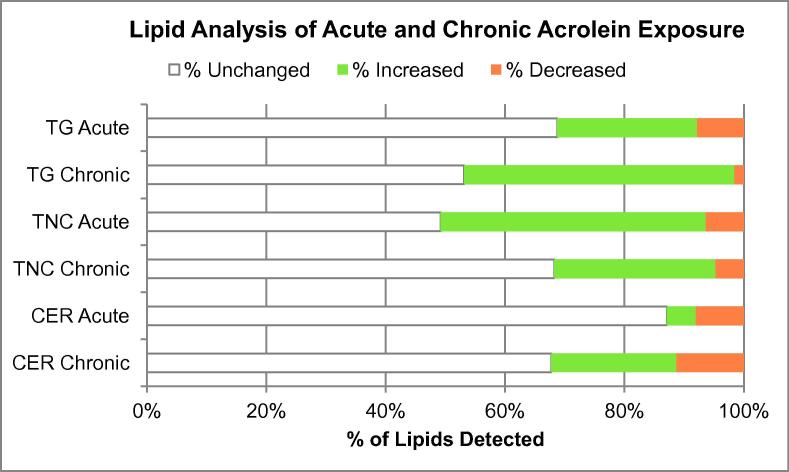
Summary of effects of acute and chronic acrolein on lipid levels in the trigeminal ganglia, trigeminal nucleus caudalis, and cerebellum. The trigeminal ganglia (TG) projects centrally to the trigeminal nucleus caudalis (TNC) in the brainstem, where sensory signals from the TG are projected onto thalamo-cortical pathways. The cerebellum (CER) was analyzed as a control tissue because it is not part of the trigeminovascular system. For each condition in each tissue type, the proportion of lipids detected that were unchanged by the acrolein exposure is shown in white. The proportion of lipids whose levels were increased by acrolein exposure in each condition is shown in green and the proportion of lipids showing a decrease relative to control is shown in orange. (For interpretation of the references to color in this figure legend, the reader is referred to the web version of this article.)

In the acute exposure paradigm (*i.e*. a single 4 h exposure to acrolein) there were varying effects on the lipidome in the TG, TNC, and CER (Fig. 1). The highest percentage of lipid changes relative to control occurred in the TNC (32 lipids – 51% of the lipids detected), followed by the TG (20 lipids – 31%), and very low amount in the CER (8 lipids – 13%). In the TG and TNC, the majority of the changes induced by acute acrolein were concentration increases relative to control. In contrast, changes detected in the CER were mostly decreases. Supplemental Fig. 5 shows the specific lipids that were affected by acrolein.

The chronic exposure paradigm (*i.e*., acrolein exposure for 4 h on 4 consecutive days) also induced changes in the lipidome in the TG, TNC, and CER (Fig. 1). The highest percentage changes occurred in the TG (30 lipids – 47% of the lipids detected), followed by the TNC and CER (both had changes in 20 lipids – 32%). Unlike in the acute paradigm, where the most changes were detected in the TNC, in the chronic paradigm the TG was the most affected tissue type (Fig. 1).

### 3.3 Effects of acrolein on *N*-acyl ethanolamines and *N*-acyl glycines

**Fig. 2:**
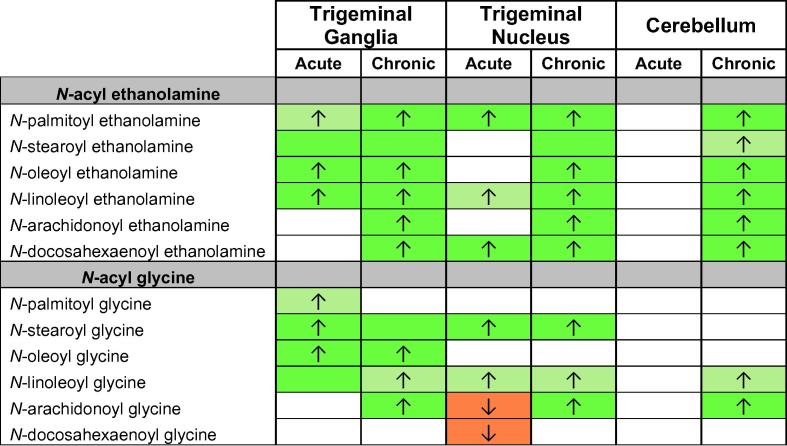
Comparison of effects of acute or chronic acrolein exposure on levels of *N*-acyl ethanolamines and *N*-acyl glycines. This figure shows changes in levels of *N*-acyl ethanolamines and *N*-acyl glycines in the trigeminal ganglia, trigeminal nucleus caudalis, and cerebellum of animals treated with acute acrolein compared to acute control and in animals treated with chronic acrolein compared to chronic control. Cells with shaded arrows indicate a change in the acrolein group relative to the control group. The arrow color indicates the direction of a significant result relative to control. Green colors represent increases, with darker green representing a significant (p < 0.05) increase and lighter green representing a trending (p < 0.1) increase. Orange colors represent decreases in a lipid’s concentration, with darker orange indicating a significant (p < 0.05) decrease and light orange representing a trending (p < 0.01) decrease. The number of arrows indicates the magnitude of the difference between the control and acrolein mice. To determine the magnitude change and therefore the number of arrows to assign each significant or trending difference, the mean level of a particular lipid in a specific region of the acrolein-treated mice was divided by that same lipid’s mean level in the same region of the corresponding control mice. For decreases the process was very similar: the mean level in the acrolein treated was divided by the mean level in the control; however, the reciprocal of the decimal was taken to express a fold decrease (if the level in the acrolein mouse is ½ of the control level then that is a 2-fold decrease). One arrow indicates a magnitude difference of less than 1.5-fold, 2 arrows indicates a 1.5–1.99-fold change, 3 arrows indicates a 2–2.99-fold change, and 4 arrows represents a magnitude difference of over 3-fold. BDL stands for “Below Detection Limit,” whereas a blank cell indicates that there was no change in the lipid’s level due to acrolein. See Methods for more detailed description of analysis. (For interpretation of the references to color in this figure legend, the reader is referred to the web version of this article.)

Acute acrolein exposure elevated levels of select *N*-acyl ethanolamines (NAEs) and had differential effects on levels of *N*-acyl glycines depending on the fatty acid conjugate (Fig. 2). In the acute paradigm, increases in *N*-palmitoyl ethanolamine (PEA), *N*-stearoyl ethanolamine, OEA, and LEA, *N*-palmitoyl glycine, *N*-stearoyl glycine (S-Gly), *N*-oleoyl glycine (O-Gly), and *N*-linoleoyl glycine (L-Gly) were seen in TG. Levels of PEA, LEA and DEA rose in the TNC. In the acute condition, levels of S-Gly and L-Gly increased, while levels of *N*-arachidonoyl glycine (NAGly) and *N*-docosahexaenoyl glycine (D-Gly) decreased in the TNC. Levels of NAEs and *N*-acyl glycines were unaffected by acute acrolein in the CER.

In the chronic paradigm the effect of acrolein on NAEs was amplified compared to the acute paradigm. For chronic acrolein exposure, levels of all 6 NAEs were increased in all three tissues (Fig. 2) as well as increases in S-Gly, O-Gly, L-Gly and NAGly. Levels of S-Gly, L-Gly and NAGly increased in the TNC. Finally, levels of L-Gly and NAGly were higher in the CER after chronic acrolein. It is noteworthy that in no case did the levels of NAEs or *N*-acyl glycines decrease in the chronic paradigm.

### 3.4 Effects of acrolein on arachidonic acid derived lipid signaling molecules

**Fig. 3:**
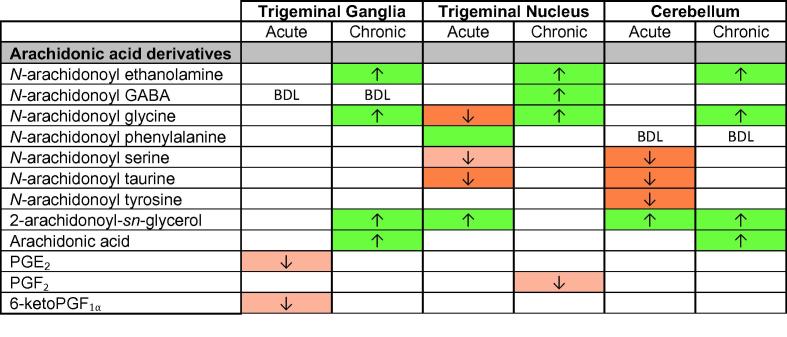
Comparison of effects of acute or chronic acrolein exposure on levels of arachidonic acid derivatives. This figure shows changes in levels of lipids derived from arachidonic acid in the trigeminal ganglia, trigeminal nucleus caudalis, and cerebellum of animals treated with acute acrolein compared to acute control and in animals treated with chronic acrolein compared to chronic control (*see*Fig 2*legend for details on shading and arrows).*

Levels of multiple lipids derived from AA in the screening library changed in the TG, TNC and CER after acute or chronic acrolein exposure (Fig. 3). Of those AA derived lipids that changed with acute acrolein the majority showed a significant decrease, which is in contrast to the overwhelming majority of all lipids that changed showing an increase. In the TG, acute acrolein levels of PGE_2_ and 6-ketoPGF_1α_ decreased, while levels of NAGly, *N*-arachidonoyl serine (A-Ser), and A-Taur decreased in the TNC during acute acrolein. In contrast, there was a striking 3-fold elevation in *N*-arachidonoyl phenylalanine (A-Phe) in the TNC as well as an in 2-AG levels. In the CER, levels of A-Ser, A-Taur, and *N*-arachidonoyl tyrosine (A-Tyr) also decreased, however levels of 2-AG increased.

After chronic exposure to acrolein, all changes in levels of AA derived lipoamines were increases relative to control (Fig. 3). Levels of AEA and NAGly were both elevated in all 3 tissue types. In addition, levels of 2-AG and free AA were raised in the TG. The CER showed the same pattern of changes as the TG. In summary, levels of AA-derived lipoamines are differentially affected by acute and chronic exposure to acrolein, mostly displaying decreases in the TNC and CER in the acute condition, and increases in all tissues in the chronic condition.

### 3.5 Effects of acrolein on levels of endogenous TRPV ligands

**Fig. 4:**
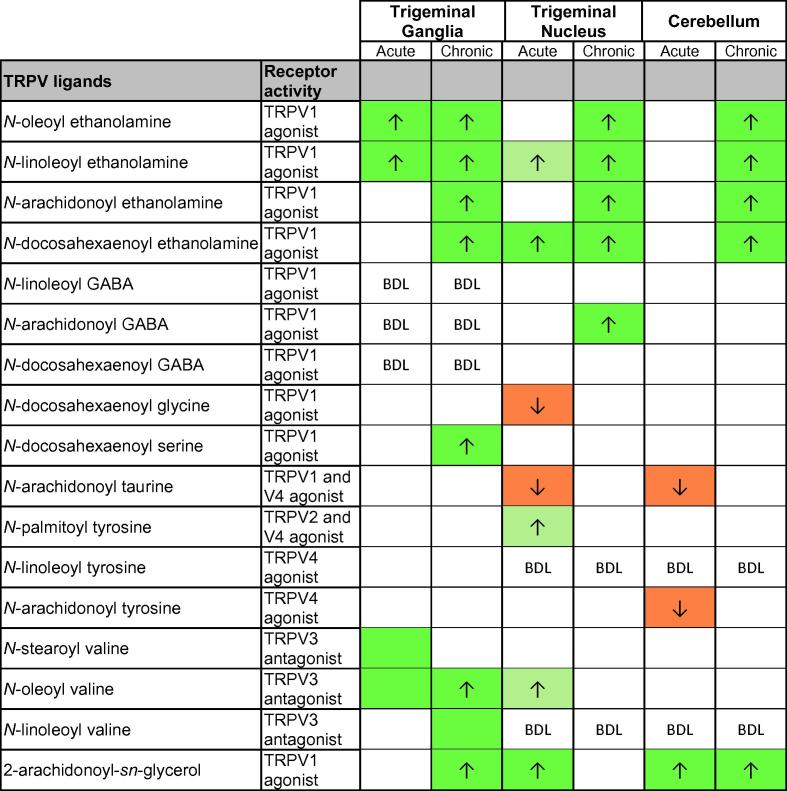
Comparison of effects of acute or chronic acrolein exposure on levels of endogenous TRPV1-4 agonists and antagonists. Some of the lipids in the screening library are endogenous ligands at TRPV1-4. This figure shows changes in levels of those lipids with activity at TRPV1-4 in the trigeminal ganglia, trigeminal nucleus caudalis, and cerebellum of animals treated with acute acrolein compared to acute control and in animals treated with chronic acrolein compared to chronic control (*see*Fig 2*legend for details on shading and arrows).*

Some of the lipids in the screening library are known ligands at TRPV1-4 (Raboune et al., 2014, Barriere et al., 2013). Of these, some were affected by both acute and chronic acrolein exposure (Fig. 4). After an acute 4 h acrolein exposure, TG levels of the TRPV1 agonists OEA and LEA increased as did levels of theTRPV3 antagonists *N*-stearoyl valine and *N*-oleoyl valine (O-Val). In the TNC, acute exposure decreased levels of the TRPV1 agonist D-Gly and the TRPV1/4 agonist A-Taur. In contrast TNC levels of some endogenous TRPV1-4 ligands increased after acute exposure, including the TRPV1 ligands LEA, DEA and 2-AG, the TRPV2/4 activator *N*-palmitoyl tyrosine, and the TRPV3 antagonist O-Val. In the CER, acute acrolein only decreased levels of 2 TRPV4 agonists (A-Taur and A-Tyr), and increased 2-AG levels.

Chronic acrolein exposure changed the levels of more TRPV ligands than did acute exposure. Furthermore, all changes in TRPV1 ligands after chronic exposure were increases. For TRPV1 ligands, most of the changes after chronic acrolein were mirrored in all 3 tissues, including increases in the TRPV1 agonists OEA, LEA, AEA and DEA. The TG also had higher levels of 2-AG and *N*-docosahexaenoyl serine following chronic acrolein exposure. Additionally, levels of the TRPV3 ligands O-Val and *N*-linoleoyl valine were exclusively increased in the TG after chronic acrolein. As well as increases in OEA, LEA, AEA and DEA, there was an increase in A-GABA in the TNC. The CER also had elevated levels of 2-AG after chronic acrolein, in addition to the increases in the NAE TRPV1 agonists. In summary, levels of at least 5 endogenous TRPV1 ligands increase in tissues of the TVS and in the CER after repeated exposure to acrolein.

### 3.6 Combinatorial activity of NAEs at TRPV1

**Fig. 5:**
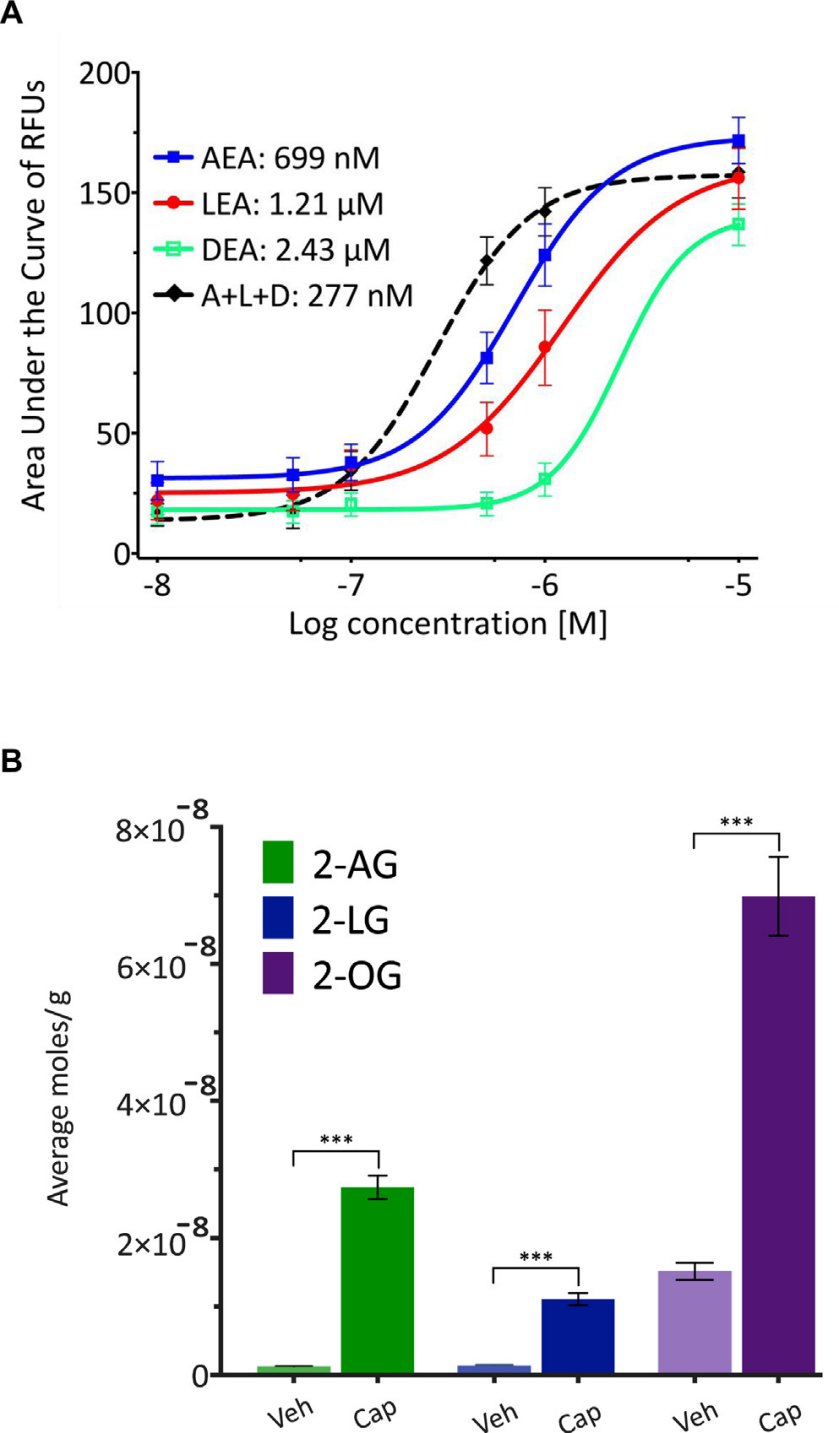
Calcium mobilization and lipid synthesis in TRV1-transfected HEK cells. A) Calcium mobilization as measured by the ratiometric dye, Fura2AM, activity. Data are the average of 4–8 wells/96-welll plate of each concentration across 6 plates. Each plate analyzed responses from compound (*N*-arachidonoyl ethanolamine: AEA; *N*-linoleoyl ethanolamine: LEA; *N*-docosahexaenoyl ethanolamine: DEA) individually and a combination of all three (A + L + D). EC_50_ values are listed to the right of the treatments in the legend. B) HPLC/MS/MS analysis of 2-acyl glycerol levels in TRPV1-HEK cells after 1 min stimulation with vehicle (0.1% DMSO) or 10 µM Capsaicin. N = 4, T-75 flasks for each condition. Data are shown as moles/gram dried pellet wt. of extracted cells.

To determine if there would be a functional consequence of an increase in these endogenous TRPV1 agonists at the receptor, we used a plate based calcium-imaging strategy in TRPV1-transfected HEK cells (Raboune et al., 2014). We challenged these cells with increasing concentrations of AEA, LEA, or DEA individually or in combination. As previously shown, each of the NAEs caused calcium mobilization with similar efficacy (Raboune et al., 2014); however, when combined there was an increase in potency of the combination reflected in an EC_50_ in the low nM range (Fig.5A). These data suggest a combinatorial effect of these lipoamines at the receptor.

### 3.7 Lipid production upon TRPV1 activation

Given that we saw significant increases in these NAE TRPV1 agonists with chronic acrolein exposure across the CNS regions analyzed, we tested the hypothesis that activation of TRPV1 drives additional lipid signaling in a manner that might explain some of the other changes in the lipidome observed with acrolein exposure. To do this we challenged TRPV1-HEK cells with 10 µM Capsaicin or vehicle and analyzed the level of NAEs and 2-acyl glycerols produced. No effect was seen on levels of NAEs (Supplemental Fig. 4B); however, levels of 3 species of 2-acyl glycerols were significantly increased with TRPV1 activation (Fig.5B). This suggests that the increases in NAEs seen after acrolein exposure are not the result of TRPV1 activation; however, the increases seen here in 2-acyl glycerols may be.

### 3.8 Effects of acrolein exposure on levels of orphan lipids

**Fig. 6:**
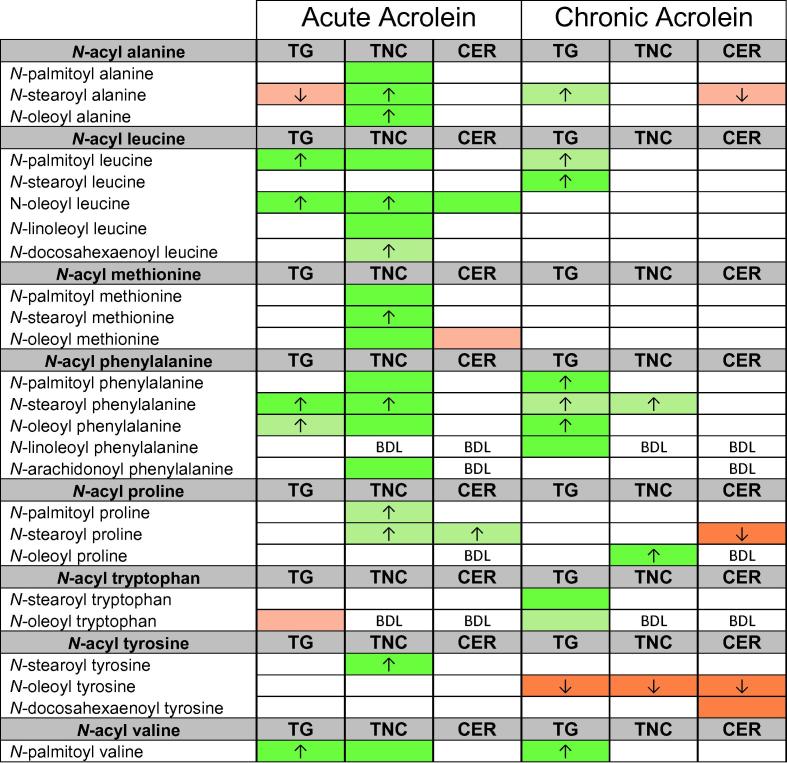
Comparison of effects of acute or chronic acrolein exposure on levels of orphan lipids. Some of the lipids in the screening library have no known protein target, and are denoted “orphan lipids.” This figure shows changes in levels of orphan lipids in the trigeminal ganglia, trigeminal nucleus caudalis, and cerebellum of animals treated with acute acrolein compared to acute control and in animals treated with chronic acrolein compared to chronic control (*see*Fig 2*legend for details on shading and arrows).*

Although their concentrations were affected by acrolein (Fig. 6), 25 lipids in the screening library have as yet no known protein target and are referred to here as orphan lipids. In the TVS, the *N*-acyl phenylalanines were upregulated by acrolein exposure. After acute acrolein exposure, levels of *N*-stearoyl phenylalanine (S-Phe) and *N*-oleoyl phenylalanine (O-Phe) increased in the TG. Similarly, in the TNC, levels of four *N*-acyl phenylalanines increased: *N*-palmitoyl phenylalanine (P-Phe), S-Phe, O-Phe and A-Phe. The increase in A-Phe in the TNC after acute acrolein was one of the largest magnitude differences, with over a 3-fold change relative to control. In the chronic paradigm, tissue specificity of the effect of acrolein on levels of *N*-acyl phenylalanines switched. The TG became the relatively more affected tissue, with changes in levels of 4 *N*-acyl phenylalanines: P-Phe, S-Phe, O-Phe and *N*-linoleoyl phenylalanine. In the TNC, only S-Phe was affected by chronic acrolein. In both the acute and chronic conditions, levels of *N*-acyl phenylalanines did not change in the CER, suggesting a specificity of signaling in the TG and TNC. Conversely, chronic acrolein exposure caused a decrease in the orphan lipid *N*-oleoyl tyrosine in all tissues and *N*-docosahexaenoyl tyrosine in CER alone, suggesting a more global regulation of these lipids with acrolein exposure.

## 4 Discussion

Exposure to acrolein, both acute and chronic, caused significant changes in lipids examined by a targeted lipidomics screen of signaling lipids centered on eCBs and TRP receptor ligands and their endogenous congeners, the lipoamines. These findings implicate acrolein as a regulator of the biosynthesis and degradation of lipid signaling molecules and demonstrate that trigeminal sensitization is accompanied by many downstream effects on lipid metabolism, potentially through TRPV1 activation. The consequences of changes in lipid levels on signaling are complicated by the fact that 25 of these lipids affected by acrolein lack a known receptor target. Exposure to acrolein is ubiquitous; it is found in air pollution and is a major component of tobacco smoke (Feng et al., 2006), vehicle exhaust fumes, some tear gasses (Brone et al., 2008), and the vapor from electronic cigarettes (Goniewicz et al., 2014). Despite the widespread effects of acrolein on lipid metabolism shown here, the dosing paradigm used in this study kept the levels of acrolein exposure below the permissible occupational health guideline of 0.3 ppm (Kunkler et al., 2015). It is important to consider that chronic exposure to low levels of acrolein can impact levels of bioactive molecules in the brain, which may create a CNS environment that can more easily transition into pathophysiology.

### 4.1 Potential role of fatty acid amide hydrolase (FAAH)

Levels of endogenous TRPV1 agonists, especially NAEs, increased after chronic exposure to acrolein. NAEs are hydrolyzed by fatty acid amide hydrolase (FAAH). Blocking FAAH significantly elevates levels of NAEs including AEA in the brain (Cravatt et al., 2001, Leishman et al., 2016b, Han et al., 2013). However, FAAH knockout (KO) mice also have lower brain levels of other AA-derived lipoamines, such as NAGly, A-GABA, and A-Taur (Leishman et al., 2016b). Therefore, an accumulation of NAE signaling molecules, as well as a decrease in NAGly, is potentially indicative of FAAH inhibition. In response to acute acrolein exposure, NAGly and A-Taur levels decreased in the TNC suggesting that some of the alterations in lipid metabolism observed in acrolein treated rats may be due to FAAH inhibition, especially in the TNC at the acute stage. We also show that the levels of A-Phe were over 3 times higher in the TNC of rats exposed to just 4 h of acrolein compared to room air controls. Unlike many of the other changes in lipid levels induced by acrolein, the change in A-Phe was very restricted to the TNC. This increase in A-Phe was the largest magnitude difference detected for any lipid measured here. However, not much is known about the actions of A-Phe in vivo. Currently, A-Phe lacks a known receptor target. One study reports that A-Phe is a mild inhibitor of FAAH (Bezuglov et al., 2006). The FAAH inhibiting activity of A-Phe may contribute to some of the altered lipoamine concentrations seen in acrolein treated rats. Follow up studies will be designed to directly examine the contribution of FAAH to the observed lipidome changes.

### 4.2 How might an accumulation of TRPV1 agonists affect pain perception?

In certain assays for acute pain, FAAH KO mice present an analgesic phenotype, hypothesized to be due AEA’s activation of CB_1_ (Cravatt et al., 2001). More recent evidence demonstrates that the phenotype of FAAH KO mice is not wholly analgesic. In response to peripheral treatment with the TRPV1 agonist capsaicin, FAAH KO mice show more nocifensive behaviors than wild-type mice (Carey et al., 2016). Similar to the effects of acrolein in this study, capsaicin treatment drove increases in levels of TRPV1 ligands in both the periphery and CNS (Carey et al., 2016). These changes in lipid signaling may underlie the increased sensitivity of FAAH KO mice to TRPV1 ligands, as TRPV1 receptors may become sensitized when constantly bombarded with much higher levels of NAEs than normal (Vellani et al., 2008). The fact that multiple NAEs were altered with acrolein exposure is important because there are emergent signaling properties when multiple TRPV1-ligand NAEs are combined (Section 3.6). The actions of multiple NAEs at TRP channels may override the analgesic effects of AEA via CB_1,_ and may explain why these changes in the lipidome sustain pro-algesic responses, rather than promoting analgesia.

### 4.3 The link between TRPV1 and 2-acyl glycerol lipids

Acrolein exposure impacted structurally analogous lipids to the eCB 2-AG. This was most apparent in the TG, where chronic acrolein raised levels of 4 different 2-acyl glycerols. What mechanisms lead to an increase in 2-acyl glycerols? Activation of TRP receptors can drive phospholipase C (PLC) intracellular pathways (Barriere et al., 2013), which stimulate the release of diacylglycerols (DAGs) from membrane phospholipids. DAGs are precursors for 2-acyl glycerols (Sugiura et al., 2006). In Section 3.7, we demonstrated that capsaicin raises levels of 2-acyl glycerols in TRPV1-HEK cells, which could potentially be due to an increase in PLC signaling. Interestingly, PLC signaling can increase trafficking of TRPA1 to the cell membrane and augments TRPA1-mediated nocifensive behavior, suggesting that PLC signaling contributes to TRPA1 sensitization (Schmidt et al., 2009). Our study did not examine TRPA1 trafficking or TRPA1 post-translational modification and the possibility that alterations in these receptor properties drive TVS sensitization cannot be excluded. However, bioactive lipids may contribute to alterations in the ability of TRP channels to respond to ligands like acrolein.

### 4.4 Peripheral inflammation drives changes in lipoamines

Using the carrageenan model of inflammatory pain (Winter et al., 1962), the Bradshaw group measured changes in brain levels of lipoamines at 1 and 3 h post-carrageenan. Notably, all brain areas tested had significant increases in NAE levels 3 h post carrageenan. The areas of the brain with the most dramatic changes in lipoamines at 3 h post carrageenan were the striatum and CER (Raboune et al., 2014). Consistent with the idea that the CER is particularly responsive to peripheral insults, lipid metabolism in the CER changes after chronic acrolein exposure. The CER was originally selected as a control tissue, as it is not part of the TVS. However, it isn’t surprising that chronic exposure to acrolein alters the lipidome in this more distant site, as the CER is in a prime location to be impacted by cross-talk between peripheral and central lipid signaling systems. Due to its many undulations, the CER has a large surface area wrapped in dural tissue. The proximity of the CER to circulating immune cells may be a source of pro-inflammatory cytokines (Garabedian et al., 2000). Although there is an absence of mRNA for TRPA1 in the CER (Saab and Nave, 2016), there is still evidence that this channel has a functional role in the CER. In cerebellar white matter, calcium influx was mediated by TRPA1 (Hamilton et al., 2016). Thus, TRPA1 may directly contribute to signaling in the CNS. Data from the carrageenan study and the current study both show that there is a time course for alterations in lipid levels in sites distant from the original insult. At just 1 h post carrageenan, there was very little effect of carrageenan treatment on the lipidome, but at 3 h, there were profound alterations in the CER. Similarly, in the present study, acrolein’s effects on lipid levels were more restricted to sites in the TVS in the acute condition, and it was in the chronic condition when the effects of acrolein on lipid levels began to fully permeate the CER.

### 4.5 Implications for migraine therapeutics

Current therapeutics for headache are not particularly effective, underscoring the need to better understand the mechanisms that cause headache and develop new drugs that target these mechanisms (Dussor et al., 2014). In this regard, TRPA1 may represent a novel target for treating migraine (Koivisto et al., 2014). Studies in the TRPA1 KO mouse (Bautista et al., 2006) suggest that TRPA1 is required for the pain and hypersensitivity that is associated with chronic inflammation. This is expected as TRPA1 functions as a gatekeeper of the release of endogenous inflammatory mediators from sensory neurons (Bautista et al., 2013). In the same rat model of acrolein-induced headache used in the present study, systemic pretreatment with the TRPA1 antagonist AP-18 prevented the sensitization of the TVS due to chronic acrolein. In acrolein-exposed rats treated with AP-18, changes in dural blood flow in response to either TRPA1 or TRPV1 agonist challenges did not sensitize the responses. The fact that responses to both types of ligands were altered by the TRPA1 blocker is important because it implicates TRPA1 in the process of sensitization regardless of the exact receptor action of the agent (Kunkler et al., 2015). Ongoing studies in our labs will test the hypothesis that TRPA1 antagonists like AP-18 may affect lipid production.

## 5 Conclusions

Both acute and chronic exposure to the environmental toxin and TRPA1 agonist acrolein causes broad, but differential, changes in the wider CNS lipidome. This broad-scale analysis of lipids in the TG, TNC and CER provide evidence that acrolein exposure drives changes in the CNS that have the potential to effect multiple signaling systems. These data indicate that there are significant differences in the outcomes of acute verses chronic TRPA1 activation and that each type of activity changes in the CNS lipidome in a different direction. Further recognition that TRP signaling systems are interconnected, dynamic, and drive the modulation multiple lipid signaling pathways will help us understand which aspects of these pathways are essential in causing CNS sensitization that may drive pain behaviors.

## Acknowledgments

*Funding:* This study was funded by the National Institutes of Health [EY024625 and DA006668 (HB) and DA024628 (EL)] and by the National Institute of Environmental Health Sciences [ES017430 (JHH and GSO)].

*Conflict of interest:* The corresponding author, Heather B Bradshaw, of this manuscript is on the Advisory Board for Phytecs and consults on how endogenous cannabinoids function in the central nervous system. Phytecs had no financial contribution to the current work.

*Ethical approval:* All animal procedures were approved by the Institutional Animal Care and Use Committee of Indiana University School of Medicine and were in accordance with the ethics guidelines set by the International Association for the Study of Pain as well as ARRIVE guidelines. This article does not contain any studies with human participants performed by any of the authors.
