# Supplementary material for: Environmental toxin acrolein alters levels of endogenous lipids, including TRP agonists: A potential mechanism for headache driven by TRPA1 activation
Source: Neurobiol Pain. 2017 Apr 1;1:28–36. doi: 10.1016/j.ynpai.2017.03.001 (PMC5802349; doi:10.1016/j.ynpai.2017.03.001)
Supplement: Supplementary data 1 [file mmc1.docx]

**Supplemental Data for Acrolein Exposure Alters Levels of Endogenous Lipids, Including TRP Agonists: A Potential Mechanism for Headache Driven by TRPA1 Activation**

**Page 3: Supplemental Figure 1** Chemical structure of acrolein

**Page 4: Supplemental Figure 2** Generic structure of a lipoamine, the conjugation of an amine and a fatty acid via an amide bond.

**Page 5: Supplemental Figure 3** Lipids in HPLC/MS/MS screening library with parent ion and fragment ion masses. Lipids are grouped by amide family and all members of that lipid family are screened in a multiple reactions monitoring (MRM) method. The parent ion mass is also listed: negative ionization mode, resulting in a [M – H]^-^ parent ion, is used for all methods except the *N*-acyl ethanolamine and 2-acyl glycerol methods, which uses positive ionization and generates a parent ion with a mass of [M + H]^+^. The parent ion is the only ion allowed to pass through the first quadrupole of the API3000. The parent ion is then fragmented into the collision chamber and an abundant fragment can be selected as the fragment ion. Only the selected fragment ion can pass from the collision chamber into the third quadrupole. Therefore, unknown lipids are matched to known standards according to retention time from the analytical column and according to their mass fingerprint.

**Page 6: Supplemental Figure 4** TRPV1-transfected HEK cells were challenged with different concentrations of combinations of *N*-linoleoyl ethanolamine (LEA), *N*- arachidonoyl ethanolamine (AEA) and *N*-docosahexaenoyl ethanolamine (DEA) and the calcium response was measured. **4A** is an illustration of the drug plate layout used for each experiment. **4B** shows the levels of NAEs measured using HPLC/MS/MS from TRPV1-HEK cells stimulated with 10uM capsaicin

**Page 7: Supplemental Figure 5:** Changes in levels of lipids in the trigeminal ganglia, trigeminal nucleus, and cerebellum of animals treated with acute acrolein compared to acute control and in animals treated with chronic acrolein compared to chronic control. Up arrows and green shading denote significant increases; whereas down arrows and orange shading denote significant decreases. See Figure 2 and Methods for more detailed description of analysis.

**Page 8: Supplemental Figure 6** Significant differences in trigeminal ganglia, trigeminal nucleus, and cerebellum lipidome in animals treated with acute acrolein compared to acute control. Only those lipids with changes from control are shown here. Up arrows and green shading denote significant increases; whereas down arrows and orange shading denote significant decreases. See Figure 2 and Methods for more detailed description of analysis.

**Page 9: Supplemental Figure 7** Significant differences in trigeminal ganglia, trigeminal nucleus, and cerebellum lipidome in animals treated with chronic acrolein compared to chronic control. Only those lipids with changes from control are shown here. Up arrows and green shading denote significant increases; whereas down arrows and orange shading denote significant decreases. See Figure 2 and Methods for more detailed description of analysis.

**Supplemental Tables are in a downloadable Excel file, each Table is on a separate sheet of a single Excel file**

All lipid levels are expressed in moles per gram of tissue. BDL = below detection limits

**Supplemental Table 1:** Lipid levels (moles per gram tissue) in the trigeminal ganglia of rats acutely treated with vehicle or acrolein

**Supplemental Table 2:** List of lipids in the trigeminal ganglia significantly affected by acute acrolein with output from statistical tests

**Supplemental Table 3:** Lipid levels (moles per gram tissue) in the trigeminal ganglia of rats chronically treated with vehicle or acrolein

**Supplemental Table 4:** List of lipids in the trigeminal ganglia significantly affected by chronic acrolein with output from statistical tests

**Supplemental Table 5:** Lipid levels (moles per gram tissue) in the trigeminal nucleus of rats acutely treated with vehicle or acrolein

**Supplemental Table 6:** List of lipids in the trigeminal nucleus significantly affected by acute acrolein with output from statistical tests

**Supplemental Table 7:** Lipid levels (moles per gram tissue) in the trigeminal nucleus of rats chronically treated with vehicle or acrolein

**Supplemental Table 8:** List of lipids in the trigeminal nucleus significantly affected by chronic acrolein with output from statistical tests

**Supplemental Table 9:** Lipid levels (moles per gram tissue) in the cerebellum of rats acutely treated with vehicle or acrolein

**Supplemental Table 10:** List of lipids in the cerebellum significantly affected by acute acrolein with output from statistical tests

**Supplemental Table 11:** Lipid levels (moles per gram tissue) in the cerebellum of rats chronically treated with vehicle or acrolein

**Supplemental Table 12:** List of lipids in the cerebellum significantly affected by chronic acrolein with output from statistical tests

**Supplemental Figure 1**

**Supplemental Figure 2**

**
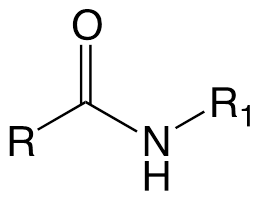
**

**R = fatty acid R_1_ = amine**

**Supplemental Figure 3**

| *N*-acyl alanine | [M – H]^-^ | Fragment |  | *N*-acyl proline | [M – H]^-^ | Fragment |
| --- | --- | --- | --- | --- | --- | --- |
| *N*-palmitoyl alanine | 326.5 | 88.09 |  | *N*-palmitoyl proline | 352.53 | 114.12 |
| *N*-stearoyl alanine | 354.55 | 88.09 |  | *N*-stearoyl proline | 380.59 | 114.12 |
| *N*-oleoyl alanine | 352.53 | 88.09 |  | *N*-oleoyl proline | 378.31 | 114.12 |
| *N*-linoleoyl alanine | 350.52 | 88.09 |  | *N*-linoleoyl proline | 376.56 | 114.12 |
| *N*-arachidonoyl alanine | 374.5 | 88.09 |  | *N*-arachidonoyl proline | 400.58 | 114.12 |
| *N*-docosahexaenoyl alanine | 398.56 | 88.09 |  | *N*-docosahexaenoyl proline | 424.6 | 114.12 |
| *N*-acyl ethanolamine | [M + H]^+^ | Fragment |  | *N*-acyl serine | [M – H]^-^ | Fragment |
| *N*-palmitoyl ethanolamine | 300.29 | 62.1 |  | *N*-palmitoyl serine | 342.3 | 74 |
| *N*-stearoyl ethanolamine | 328.3 | 62.1 |  | *N*-stearoyl serine | 370.3 | 74 |
| *N*-oleoyl ethanolamine | 326.3 | 62.1 |  | *N*-oleoyl serine | 368.3 | 74 |
| *N*-linoleoyl ethanolamine | 324.3 | 62.1 |  | *N*-linoleoyl serine | 366.27 | 74 |
| *N*-arachidonoyl ethanolamine | 348.29 | 62.1 |  | *N*-arachidonoyl serine | 390.3 | 74 |
| *N*-docosahexaenoyl ethanolamine | 372.6 | 62.1 |  | *N*-docosahexaenoyl serine | 414.3 | 74 |
| *N*-acyl GABA | [M – H]^-^ | Fragment |  | *N*-acyl taurine | [M – H]^-^ | Fragment |
| *N*-palmitoyl GABA | 340.54 | 102.1 |  | *N*-arachidonoyl taurine | 410.6 | 124 |
| *N*-stearoyl GABA | 368.58 | 102.1 |  | *N*-acyl tryptophan | [M – H]^-^ | Fragment |
| *N*-oleoyl GABA | 366.57 | 102.1 |  | *N*-palmitoyl tryptophan | 441.63 | 203.1 |
| *N*-linoleoyl GABA | 364.54 | 102.1 |  | *N*-stearoyl tryptophan | 469.68 | 203.1 |
| *N*-arachidonoyl GABA | 388.57 | 102.1 |  | *N*-oleoyl tryptophan | 467.67 | 203.1 |
| *N*-docosahexaenoyl GABA | 412.59 | 102.1 |  | *N*-linoleoyl tryptophan | 465.65 | 203.1 |
| *N*-acyl glycine | [M – H]^-^ | Fragment |  | *N*-arachidonoyl tryptophan | 489.67 | 203.1 |
| *N*-palmitoyl glycine | 312.26 | 74.2 |  | *N*-docosahexaenoyl tryptophan | 513.69 | 203.1 |
| *N*-stearoyl glycine | 340.3 | 74.2 |  | *N*-acyl tyrosine | [M – H]^-^ | Fragment |
| *N*-oleoyl glycine | 338.3 | 74.2 |  | *N*-palmitoyl tyrosine | 418.59 | 180.18 |
| *N*-linoleoyl glycine | 336.3 | 74.2 |  | *N*-stearoyl tyrosine | 446.65 | 180.18 |
| *N*-arachidonoyl glycine | 360.3 | 74.2 |  | *N*-oleoyl tyrosine | 444.63 | 180.18 |
| *N*-docosahexaenoyl glycine | 384.3 | 74.2 |  | *N*-linoleoyl tyrosine | 442.61 | 180.18 |
| *N*-acyl leucine | [M – H]^-^ | Fragment |  | *N*-arachidonoyl tyrosine | 466 | 180.18 |
| *N*-palmitoyl leucine | 368.58 | 130.1 |  | *N*-docosahexaenoyl tyrosine | 490.66 | 180.18 |
| *N*-stearoyl leucine | 396.63 | 130.1 |  | *N*-acyl valine | [M – H]^-^ | Fragment |
| *N*-oleoyl leucine | 394.61 | 130.1 |  | *N*-palmitoyl valine | 354.31 | 116.31 |
| *N*-linoleoyl leucine | 392.6 | 130.1 |  | *N*-stearoyl valine | 382.6 | 116.14 |
| *N*-docosahexaenoyl leucine | 440.64 | 130.1 |  | *N*-oleoyl valine | 380.59 | 116.14 |
| *N*-acyl methionine | [M – H]^-^ | Fragment |  | *N*-linoleoyl valine | 378.58 | 116.14 |
| *N*-palmitoyl methionine | 386.62 | 148.2 |  | *N*-docosahexaenoyl valine | 426.62 | 116.14 |
| *N*-stearoyl methionine | 414.64 | 148.2 |  | Free Fatty Acids | [M – H]^-^ | Fragment |
| *N*-oleoyl methionine | 412.65 | 148.2 |  | Linoleic Acid | 279.5 | 261 |
| *N*-linoleoyl methionine | 410.64 | 148.2 |  | Arachidonic Acid | 303.5 | 285 |
| *N*-arachidonoyl methionine | 434.66 | 148.2 |  | 2-acyl-*sn*-glycerol | [M + H]^+^ | Fragment |
| *N*-docosahexaenoyl methionine | 458.68 | 148.2 |  | 2-arachidonoyl-*sn*-glycerol | 379.3 | 287.5 |
| *N*-acyl phenylalanine | [M – H]^-^ | Fragment |  | 2-linoleoyl-*sn*-glycerol | 355.5 | 245 |
| *N*-palmitoyl phenylalanine | 402.59 | 164.1 |  | 2-oleoyl-*sn*-glycerol | 357.5 | 265.2 |
| *N*-stearoyl phenylalanine | 430.65 | 164.1 |  | 2-palmitoyl-*sn*-glycerol | 331.5 | 239.5 |
| *N*-oleoyl phenylalanine | 428.63 | 164.1 |  | Prostaglandins | [M – H]^-^ | Fragment |
| *N*-linoleoyl phenylalanine | 426.61 | 164.1 |  | PGE_2_ | 351.2 | 315 |
| *N*-arachidonoyl phenylalanine | 450.64 | 164.1 |  | PGF_2α_ | 353.3 | 309.2 |
| *N*-docosahexaenoyl phenylalanine | 474.66 | 164.1 |  | 6-ketoPGF_1α_ | 369.3 | 206.9 |

**
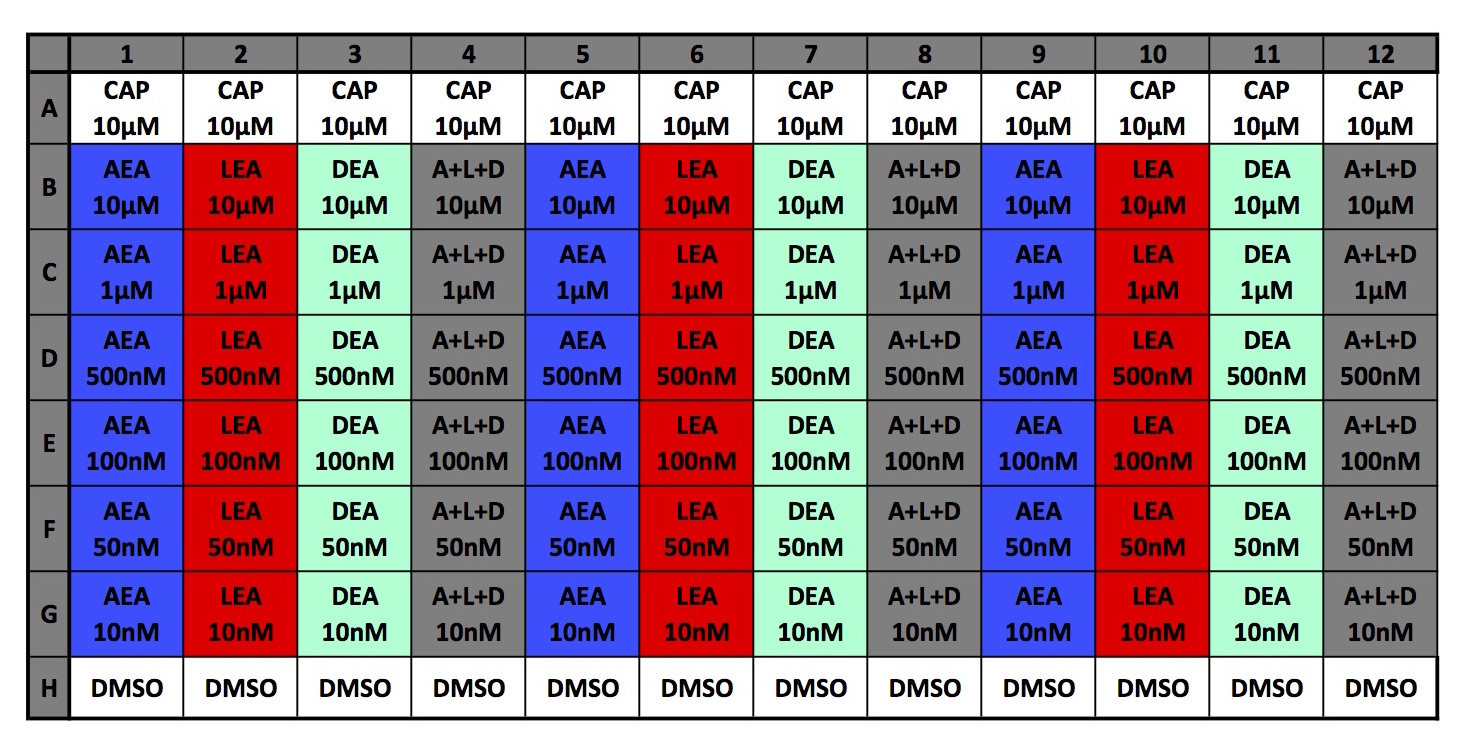
Supplemental Figure 4**

**Supplemental Figure 4A.** Assay conditions for calcium imaging of independent and combined NAEs.

**Supplemental Figure 5**

|  | Trigeminal Ganglia | | Trigeminal Nucleus | | Cerebellum | |
| --- | --- | --- | --- | --- | --- | --- |
| ***N*-acyl alanine** | Acute | Chronic | Acute | Chronic | Acute | Chronic |
| *N*-palmitoyl alanine |  |  | ↑↑ |  |  |  |
| *N*-stearoyl alanine | ↓ | ↑ | ↑ |  |  | ↓ |
| *N*-oleoyl alanine |  |  | ↑ |  |  |  |
| ***N*-acyl ethanolamine** | Acute | Chronic | Acute | Chronic | Acute | Chronic |
| *N*-palmitoyl ethanolamine | ↑ | ↑ | ↑ | ↑ |  | ↑ |
| *N*-stearoyl ethanolamine | ↑↑ | ↑↑ |  | ↑↑ |  | ↑ |
| *N*-oleoyl ethanolamine | ↑ | ↑ |  | ↑ |  | ↑ |
| *N*-linoleoyl ethanolamine | ↑ | ↑ | ↑ | ↑ |  | ↑ |
| *N*-arachidonoyl ethanolamine |  | ↑ |  | ↑ |  | ↑ |
| *N*-docosahexaenoyl ethanolamine |  | ↑ | ↑ | ↑ |  | ↑ |
| ***N*-acyl GABA** | Acute | Chronic | Acute | Chronic | Acute | Chronic |
| *N*-palmitoyl GABA |  |  | ↑ | ↑ |  |  |
| *N*-stearoyl GABA |  |  |  | ↑ |  |  |
| *N*-oleoyl GABA | BDL | BDL |  | ↑ | ↓ | ↓ |
| *N*-arachidonoyl GABA | BDL | BDL |  | ↑ |  |  |
| ***N*-acyl glycine** | Acute | Chronic | Acute | Chronic | Acute | Chronic |
| *N*-palmitoyl glycine | ↑ |  |  |  |  |  |
| *N*-stearoyl glycine | ↑ | ↑↑ | ↑ | ↑ |  |  |
| *N*-oleoyl glycine | ↑ | ↑ |  |  |  |  |
| *N*-linoleoyl glycine | ↑↑ | ↑ | ↑ | ↑ |  | ↑ |
| *N*-arachidonoyl glycine |  | ↑ | ↓ | ↑ |  | ↑ |
| *N*-docosahexaenoyl glycine |  |  | ↓ |  |  |  |
| ***N*-acyl leucine** | Acute | Chronic | Acute | Chronic | Acute | Chronic |
| *N*-palmitoyl leucine | ↑ | ↑ | ↑↑ |  |  |  |
| *N*-stearoyl leucine |  | ↑ |  |  |  |  |
| *N*-oleoyl leucine | ↑ |  | ↑ |  | ↑↑ |  |
| *N*-linoleoyl leucine |  |  | ↑↑ |  |  |  |
| *N*-docosahexaenoyl leucine |  |  | ↑ |  |  |  |
| ***N*-acyl methionine** | Acute | Chronic | Acute | Chronic | Acute | Chronic |
| *N*-palmitoyl methionine |  |  | ↑↑↑ |  |  |  |
| *N*-stearoyl methionine |  |  | ↑ |  |  |  |
| *N*-oleoyl methionine |  |  | ↑↑↑ |  | ↓↓↓ |  |
| ***N*-acyl phenylalanine** | Acute | Chronic | Acute | Chronic | Acute | Chronic |
| *N*-palmitoyl phenylalanine |  | ↑ | ↑↑↑ |  |  |  |
| *N*-stearoyl phenylalanine | ↑ | ↑ | ↑ | ↑ |  |  |
| *N*-oleoyl phenylalanine | ↑ | ↑ | ↑↑ |  |  |  |
| *N*-linoleoyl phenylalanine |  | ↑↑↑ | BDL | BDL | BDL | BDL |
| *N*-arachidonoyl phenylalanine |  |  | ↑↑↑↑ |  | BDL | BDL |
| ***N*-acyl proline** | Acute | Chronic | Acute | Chronic | Acute | Chronic |
| *N*-palmitoyl proline |  |  | ↑ |  |  |  |
| *N*-stearoyl proline |  |  | ↑ |  | ↑ | ↓ |
| *N-*oleoyl proline |  |  |  | ↑ | BDL | BDL |
| ***N*-acyl serine** | Acute | Chronic | Acute | Chronic | Acute | Chronic |
| *N*-palmitoyl serine | ↓ |  |  |  |  |  |
| *N*-oleoyl serine |  |  |  |  |  | ↓ |
| *N*-linoleoyl serine |  |  |  | ↓↓ |  | ↓ |
| *N*-arachidonoyl serine |  |  | ↓ |  | ↓ |  |
| *N*-docosahexaenoyl serine |  | ↑ |  |  |  |  |
| ***N*-acyl taurine** | Acute | Chronic | Acute | Chronic | Acute | Chronic |
| *N*-arachidonoyl taurine |  |  | ↓ |  | ↓ |  |
| ***N*-acyl tryptophan** | Acute | Chronic | Acute | Chronic | Acute | Chronic |
| *N*-stearoyl tryptophan |  | ↑↑ |  |  |  |  |
| *N*-oleoyl tryptophan | ↓↓↓ | ↑↑↑ | BDL | BDL | BDL | BDL |
| ***N*-acyl tyrosine** | Acute | Chronic | Acute | Chronic | Acute | Chronic |
| *N*-palmitoyl tyrosine |  |  | ↑ |  |  |  |
| *N*-stearoyl tyrosine |  |  | ↑ |  |  |  |
| *N-*oleoyl tyrosine |  | ↓ |  | ↓ |  | ↓ |
| *N*-arachidonoyl tyrosine |  |  |  |  | ↓ |  |
| *N*-docosahexaenoyl tyrosine |  |  |  |  |  | ↓↓↓ |
| ***N*-acyl valine** | Acute | Chronic | Acute | Chronic | Acute | Chronic |
| *N*-palmitoyl valine | ↑ | ↑ | ↑↑ |  |  |  |
| *N*- stearoyl valine | ↑↑ |  |  |  |  |  |
| *N*-oleoyl valine | ↑↑ | ↑ | ↑ |  |  |  |
| *N*-linoleoyl valine |  | ↑↑ | BDL | BDL | BDL | BDL |
| **Free Fatty Acids** | Acute | Chronic | Acute | Chronic | Acute | Chronic |
| Linoleic acid |  | ↑ |  |  |  | ↑ |
| Arachidonic acid |  | ↑ |  |  |  | ↑ |
| **2-acyl glycerol** | Acute | Chronic | Acute | Chronic | Acute | Chronic |
| 2-arachidonoyl glycerol |  | ↑ | ↑ |  | ↑ | ↑ |
| 2-linoleoyl glycerol |  | ↑ | ↑ | ↑ |  | ↑ |
| 2-oleoyl glycerol |  | ↑↑ |  | ↑ |  | ↑ |
| 2-palmitoyl glycerol |  | ↑ |  |  |  |  |
| **Prostaglandins** | Acute | Chronic | Acute | Chronic | Acute | Chronic |
| PGE_2_ | ↓ |  |  |  |  |  |
| PGF_2α_ |  |  |  | ↓ |  |  |
| 6-ketoPGF_1α_ | ↓ |  |  |  |  |  |

**Supplemental Figure 6: Effects of acute acrolein on lipid levels**

|  | **Trigeminal Ganglia** | **Trigeminal Nucleus** | **Cerebellum** |
| --- | --- | --- | --- |
| ***N*-acyl alanine** |  |  |  |
| *N*-palmitoyl alanine |  | ↑↑ |  |
| *N*-stearoyl alanine | ↓ | ↑ |  |
| *N*-oleoyl alanine |  | ↑ |  |
| ***N*-acyl ethanolamine** |  |  |  |
| *N*-palmitoyl ethanolamine | ↑ | ↑ |  |
| *N*-stearoyl ethanolamine | ↑↑ |  |  |
| *N*-oleoyl ethanolamine | ↑ |  |  |
| *N*-linoleoyl ethanolamine | ↑ | ↑ |  |
| *N*-docosahexaenoyl ethanolamine |  | ↑ |  |
| ***N*-acyl GABA** |  |  |  |
| *N*-palmitoyl GABA |  | ↑ |  |
| *N*-oleoyl GABA | BDL |  | ↓ |
| ***N*-acyl glycine** |  |  |  |
| *N*-palmitoyl glycine | ↑ |  |  |
| *N*-stearoyl glycine | ↑ | ↑ |  |
| *N*-oleoyl glycine | ↑ |  |  |
| *N*-linoleoyl glycine | ↑↑ | ↑ |  |
| *N*-arachidonoyl glycine |  | ↓ |  |
| *N*-docosahexaenoyl glycine |  | ↓ |  |
| ***N*-acyl leucine** |  |  |  |
| *N*-palmitoyl leucine | ↑ | ↑↑ |  |
| *N*-oleoyl leucine | ↑ | ↑ | ↑↑ |
| *N*-linoleoyl leucine |  | ↑↑ |  |
| *N*-docosahexaenoyl leucine |  | ↑ |  |
| ***N*-acyl methionine** |  |  |  |
| *N*-palmitoyl methionine |  | ↑↑↑ |  |
| *N*-stearoyl methionine |  | ↑ |  |
| *N*-oleoyl methionine |  | ↑↑↑ | ↓↓↓ |
| ***N*-acyl phenylalanine** |  |  |  |
| *N*-palmitoyl phenylalanine |  | ↑↑↑ |  |
| *N*-stearoyl phenylalanine | ↑ | ↑ |  |
| *N*-oleoyl phenylalanine | ↑ | ↑↑ |  |
| *N*-arachidonoyl phenylalanine |  | ↑↑↑↑ | BDL |
| ***N*-acyl proline** |  |  |  |
| *N*-palmitoyl proline |  | ↑ |  |
| *N*-stearoyl proline |  | ↑ | ↑ |
| ***N*-acyl serine** |  |  |  |
| *N*-palmitoyl serine | ↓ |  |  |
| *N*-arachidonoyl serine |  | ↓ | ↓ |
| ***N*-acyl taurine** |  |  |  |
| *N*-arachidonoyl taurine |  | ↓ | ↓ |
| ***N*-acyl tryptophan** |  |  |  |
| *N*-oleoyl tryptophan | ↓↓↓ | BDL | BDL |
| ***N*-acyl tyrosine** |  |  |  |
| *N*-palmitoyl tyrosine |  | ↑ |  |
| *N*-stearoyl tyrosine |  | ↑ |  |
| *N*-arachidonoyl tyrosine |  |  | ↓ |
| ***N*-acyl valine** |  |  |  |
| *N*-palmitoyl valine | ↑ | ↑↑ |  |
| *N*- stearoyl valine | ↑↑ |  |  |
| *N*-oleoyl valine | ↑↑ | ↑ |  |
| **2-acyl-*sn*-glycerol** |  |  |  |
| 2-arachidonoyl-*sn*-glycerol |  | ↑ | ↑ |
| 2-linoleoyl-*sn*-glycerol |  | ↑ |  |
| **Prostaglandins** |  |  |  |
| PGE_2_ | ↓ |  |  |
| 6-ketoPGF_1α_ | ↓ |  |  |

**Supplemental Figure 7: Effects of chronic acrolein on lipid levels**

|  | **Trigeminal Ganglia** | **Trigeminal Nucleus** | **Cerebellum** |
| --- | --- | --- | --- |
| ***N*-acyl alanine** |  |  |  |
| *N*-stearoyl alanine | ↑ |  | ↓ |
| ***N*-acyl ethanolamine** |  |  |  |
| *N*-palmitoyl ethanolamine | ↑ | ↑ | ↑ |
| *N*-stearoyl ethanolamine | ↑↑ | ↑↑ | ↑ |
| *N*-oleoyl ethanolamine | ↑ | ↑ | ↑ |
| *N*-linoleoyl ethanolamine | ↑ | ↑ | ↑ |
| *N*-arachidonoyl ethanolamine | ↑ | ↑ | ↑ |
| *N*-docosahexaenoyl ethanolamine | ↑ | ↑ | ↑ |
| ***N*-acyl GABA** |  |  |  |
| *N*-palmitoyl GABA |  | ↑ |  |
| *N*-stearoyl GABA |  | ↑ |  |
| *N*-oleoyl GABA | BDL | ↑ | ↓ |
| *N*-arachidonoyl GABA | BDL | ↑ |  |
| ***N*-acyl glycine** |  |  |  |
| *N*-stearoyl glycine | ↑↑ | ↑ |  |
| *N*-oleoyl glycine | ↑ |  |  |
| *N*-linoleoyl glycine | ↑ | ↑ | ↑ |
| *N*-arachidonoyl glycine | ↑ | ↑ | ↑ |
| ***N*-acyl leucine** |  |  |  |
| *N*-palmitoyl leucine | ↑ |  |  |
| *N*-stearoyl leucine | ↑ |  |  |
| ***N*-acyl phenylalanine** |  |  |  |
| *N*-palmitoyl phenylalanine | ↑ |  |  |
| *N*-stearoyl phenylalanine | ↑ | ↑ |  |
| *N*-oleoyl phenylalanine | ↑ |  |  |
| *N*-linoleoyl phenylalanine | ↑↑↑ | BDL | BDL |
| ***N*-acyl proline** |  |  |  |
| *N*-stearoyl proline |  |  | ↓ |
| *N-*oleoyl proline |  | ↑ | BDL |
| ***N*-acyl serine** |  |  |  |
| *N*-oleoyl serine |  |  | ↓ |
| *N*-linoleoyl serine |  | ↓↓ | ↓ |
| *N*-docosahexaenoyl serine | ↑ |  |  |
| ***N*-acyl tryptophan** |  |  |  |
| *N*-stearoyl tryptophan | ↑↑ |  |  |
| *N*-oleoyl tryptophan | ↑↑↑ | BDL | BDL |
| ***N*-acyl tyrosine** |  |  |  |
| *N-*oleoyl tyrosine | ↓ | ↓ | ↓ |
| *N*-docosahexaenoyl tyrosine |  |  | ↓↓↓ |
| ***N*-acyl valine** |  |  |  |
| *N*-palmitoyl valine | ↑ |  |  |
| *N*-oleoyl valine | ↑ |  |  |
| *N*-linoleoyl valine | ↑↑ | BDL | BDL |
| **Free Fatty Acids** |  |  |  |
| Linoleic acid | ↑ |  | ↑ |
| Arachidonic acid | ↑ |  | ↑ |
| **2-acyl-*sn*-glycerol** |  |  |  |
| 2-arachidonoyl-*sn*-glycerol | ↑ |  | ↑ |
| 2-linoleoyl-*sn*-glycerol | ↑ | ↑ | ↑ |
| 2-oleoyl-*sn*-glycerol | ↑↑ | ↑ | ↑ |
| 2-palmitoyl glycerol | ↑ |  |  |
| **Prostaglandins** |  |  |  |
| PGF_2α_ |  | ↓ |  |
